# A neuro-cognitive model of comprehension based on prediction and unification

**DOI:** 10.3389/fnhum.2024.1356541

**Published:** 2024-04-09

**Authors:** Philippe Blache

**Affiliations:** ^1^Laboratoire Parole et Langage (LPL-CNRS), Aix-en-Provence, France; ^2^Institute of Language, Communication and the Brain (ILCB), Marseille, France

**Keywords:** neuro-cognitive model, unification, prediction, facilitation, language processing

## Abstract

Most architectures and models of language processing have been built upon a restricted view of language, which is limited to sentence processing. These approaches fail to capture one primordial characteristic: efficiency. Many facilitation effects are known to be at play in natural situations such as conversation (shallow processing, no real access to the lexicon, etc.) without any impact on the comprehension. In this study, on the basis of a new model integrating into a unique architecture, we present these facilitation effects for accessing the meaning into the classical compositional architecture. This model relies on two mechanisms, prediction and unification, and provides a unique architecture for the description of language processing in its natural environment.

## 1 Introduction

During natural conversations, language is produced very rapidly, approximately 3–5 words per second, sometimes even faster. This means that the amount of time allocated for producing and perceiving a word in a natural situation should be approximately 2–300 ms per word. However, when exploring the time course of language perception in the brain, we observe effects at different stages from 200 ms (lexical access, early effects in morpho-syntactic violations etc.) to 600–800 ms or even more for integrating a new word into the interpretation under construction (Friederici, 2002; Kaan, 2007). A long latency is also observed for production, generating the phonological form of a word follows different stages from 180 to 450 ms (Indefrey, 2011; Strijkers and Costa, 2011). Therefore, there is a significant gap between what is predicted by theory in neurolinguistics, mainly based on laboratory experiments (up to 1 s processing per word) and what is actually observed in natural situation both for production and perception (2–300 ms per word). Of course, parallelism and overlap have been shown to play a central role (Huettig et al., 2022), explaining part of this gap, as well as buffering mechanisms (Vagharchakian et al., 2012). However, more importantly, several facilitation mechanisms can explain the fact that language is processed in real time, with no delay. Exploring this issue requires to answer two questions: (1) What are the mechanisms that can speed up the processing and (2) How are they involved in a global processing architecture? The main goal of this study is to propose a model of language processing integrating such facilitation mechanisms.

Linguistic theories and computational and neuro-cognitive models rely on the idea that linguistic information is organized into different levels, usually processed in a serial way, in relation with the sequential nature of the input signal. In this type of organization, each level takes as input an information coming from the level below (completed in some cases with other contextual information). This architecture is thus hierarchical from lower levels to abstract representations of the meaning (from phonetics to semantics), each level operating as a function taking an input and returning an output representation.

However, several studies have shown that one or more levels can be skipped in many situations. For example, in the case of idiomatic constructions, there is no trace in the brain signal of an access to the lexical meaning after the recognition point of the idiom (Rommers et al., 2013; Vulchanova et al., 2019; Kessler et al., 2021). The meaning of the remaining words of the idiom after this point is no more activated; the entire idiom has been retrieved from the memory, the only processing consists in verifying the match between the form of the idiom and the words produced by the speaker. In case of mismatch, the related brain activity evokes a reaction similar to a morphological error (Rommers et al., 2013).

In a similar way, the syntactic structure can be by-passed, for example, in the case of *semantic attraction* (Kuperberg, 2007; Brouwer et al., 2017), typically a situation of mismatch between grammatical and thematic roles. In the same vein, many studies in the framework of good-enough theory (Ferreira et al., 2002) have also shown that a shallow processing is often at work, avoiding building an exact syntactic structure. Note that for their part, several linguistic theories also propose the idea that language processing is based on the identification of an optimal structure, possibly ill-formed and partial, challenging the idea of a strict hierarchical organization (Prince and Smolensky, 1993; Blache, 2016).

Generally speaking, situations where processing can be accelerated are extremely frequent, especially during conversation. It is thus necessary to integrate such facilitation mechanisms into a general architecture of language processing. However, to date, no clear answer has been proposed by existing models for integrating fuzziness, shortcuts, or partial information. Moreover, it is difficult to imagine that two different types of processes cohabit: one that would correspond to the classical hierarchical organization, the other implementing facilitation mechanisms and shortcuts skipping one or more linguistic levels to directly access the meaning.

This study addresses this question by proposing the basis of a unique architecture bringing together both facilitation and standard processing mechanisms. This proposal relies on several ideas:

- The basic processing components can be of different granularities, from partial and underspecified objects to words, constructions or entire pieces of knowledge;
- These units can be predicted, whatever their granularity;
- They can integrate information from the different linguistic domains (and can be multimodal);
- The integration mechanism is based on the comparison of what is predicted with what is perceived;
- Comprehension consists in updating a global model, at the level of the conversation or the text;

In our model, instead of considering that two concurrent mechanisms are in competition, one being hierarchical and the other not, we propose to distinguish between cases where the linguistic objects to be processed can be at a high or low level of integration; in some cases, objects can correspond to single words or, in other cases, to complex constructions, encoding a complete piece of meaning. As it has been described in many neuro-cognitive paradigms, the processing is hierarchical in the sense that goes from lower to higher levels of abstraction (Ryskin and Nieuwland, 2023); in the classical description, it goes from the phonemes to the meaning. Note that this mechanism is parallel in the sense that each domain (phonetics, syntax, semantics, etc.) can work more or less independently (Jackendoff, 2007; Baggio, 2021; Huettig et al., 2022). In our model, we propose to still consider processing as hierarchical by integrating a structure into a more complex one. However, in some cases, the processing starts based on high-level complex objects, already bearing a lot of integrated information from many different sources or domains. In such cases, the information usually resulting from the processing of lower level is already compiled into the structure.

In this study, we present the mechanisms implementing the complete architecture and describe in detail the processing cycle.

## 2 How to access the meaning? A theoretical perspective

We propose to start with two preliminary questions: What are the mechanisms at play when accessing the meaning and What are the basic components. We address these questions through the prism of two theoretical frameworks from linguistics and cognitive neuroscience: 1/ *Construction Grammars* and how they explain a direct access to the meaning; 2/ *Memory, Unification, and Control* and how unification renders possible the use of complex basic components encoding entire pieces of meaning.

### 2.1 Direct access to the meaning: construction vs. compositionality

Many consider that communication is the main purpose of language. This means that the goal of a language processing model should be to explain how people understand each other by communicating with language (keeping in mind that language is a means among others to communicate). In more formal terms, understanding means accessing the meaning. In classical theories, this process is based on the *compositionality principle* stipulating that the meaning of a complex expression is a function of the meanings of its parts (see Dowty, 2007 for a global discussion and Szabó, 2004 for a formal overview). Understanding a sentence (sentence being usually the level considered in formal semantics) consists in proceeding step-by-step, bottom-up, by first accessing the meaning of words, then aggregating these meanings into larger structures until reaching the meaning of the sentence. The syntactic structure is at the core of the composition function by identifying the different sub-parts to combine and their relations. This mechanism is based on the hypothesis of the existence a homomorphism, mapping the syntactic algebra into the semantic algebra (Montague, 1973).

However, several studies have shown that accessing the meaning can also be direct in a top-down manner. In particular, *Construction Grammar* (Fillmore, 1988) is based on the idea that language structures are constructions made of *form/meaning* pairings, offering direct access to the meaning, thus calling into question the compositional mechanism (Goldberg, 2016). Idioms are such typical constructions: when the idiom is recognized (usually after few words), its (figurative) meaning is activated, without needing to analyze the rest of the input. The same mechanism is at work for all constructions: a direct access to the construction meaning is possible when the form of the construction is recognized.

The question is to explain how both routes, compositional and direct, for accessing the meaning co-exist.

### 2.2 Memory and unification

The *Memory, Unification, and Control* model (hereafter MUC) relies on different ideas challenging what is classically integrated in other neuro-cognitive models (Hagoort, 2005, 2013). First, MUC is based on linguistic representations much more intricate than usually considered: the basic units (the items stored in the mental lexicon) contain rich information at different levels: phonology, morphology, semantics, and syntax. The question of what is stored in the memory is therefore a critical issue. The idea of having high-level linguistic information (including partial structures) already compiled in the memory represent the first and maybe the most important facilitation mechanism (Baggio and Hagoort, 2011). It has moreover a direct consequence on the processing architecture; the mechanism consists in simply aggregating this high-level components, integrating directly complex pieces of information. It offers at the same time a way to implement a fine control of the processing by providing multiple information from different sources, offering first possibility to make precise prediction about what is expected and second a mean to precisely compare what is expected with what is realized. MUC integrates these aspects by distinguishing three different components: *memory, unification*, and *control*.

*Memory* corresponds to a repository storing all information associated with basic units. Following a proposal called *lexicalization* initiated by constraint-based linguistic theories such as Head-Driven Phrase Structure Grammar (Pollard and Sag, 1994), Tree-Adjoining Grammar (Joshi and Schabes, 1997), or Lexical-Functional Grammar (Bresnan, 1982), lexical entries encode high-level information, possibly concerning phonology, morphology, syntax, and semantics.^1^ In these approaches, parsing an input does not consist anymore in building a tree but in aggregating the basic units due to a mechanism called *unification* (see below). The initial inspiration in MUC was thus to encode in the *Memory* lexical units associated with partial trees as proposed in TAG and then enrich these units with different information as it is the case with constraint-based theories (Sag, 2012). This idea of bringing together different sources of information, from different domains and modalities, has been pushed forward with Construction Grammars (Fillmore, 1998). In this theory, the basic components are constructions that can represent lexical structures and complex patterns (also called MUC *syntactic frames*), including, for example, long-distance dependencies. *Memory* in MUC refers to such complex units that are stored and retrieved from the long-term memory.

The *Unification* component takes in charge the question of how to assemble basic units. The key aspect is that units stored in the memory are associated with constraints that are mainly stipulated in terms of restrictions over feature values (e.g., verbs usually impose some semantic characteristics on their arguments). Unification is a mathematical operation, which is intensively used in linguistics since the origin of Unification Grammars (Kay, 1979). It basically offers the possibility to evaluate the compatibility between two structures (see Section 4.1). Consequently, the syntactic analysis mechanism consists of checking the compatibility between a predicted structure and the input using unification only (getting rid of derivation rules). We present this mechanism in more details in Section 4.

The third component, called *Control*, is connected with discourse-level phenomena, joint action, social interaction, context, and more generally pragmatics. This component might be in relation with attentional mechanisms that can be triggered by specific linguistic devices (e.g., focus accent) in order to mark important features.

A *processing cycle* is described in MUC on the basis of the connectivity between the different regions involved in this architecture (Baggio and Hagoort, 2011). Starting from the sensory regions (visual or audio depending on the input modality), the signal is conveyed to the regions where lexical information is activated (inferior, middle, and superior temporal gyri: the posterior temporal regions). All associated constraints but also information coming from other modalities are collected there; this is the role of the *Memory* component. A local activation within temporal areas propagates information and activates related lexical structures, building the semantic context in temporal cortex. From these regions, the activated structures are passed to the inferior frontal regions, hosting *Unification*. The information activated there is propagated back to the temporal regions, forming a cycle. This back-propagation triggers another spread of activation to neighboring temporal areas, updating the semantic context. What is interesting in this processing cycle is that the architecture is not anymore strictly sequential but leaves place to cycles implementing an interaction between *Memory* and *Unification*. On top of checking the compliance of the activated and predicted structures, this organization explains how units from different levels (lexical or complex constructions) can be accessed within the same cycle.

One of the major contributions of MUC has been to provide evidence at the brain level in favor of a unique language processing mechanism: unification. On top of being a crucial argument supporting constraint-based linguistic theories, MUC also constitutes a specific neuro-cognitive approach to language processing putting aside at the same time the classical rule-based compositional approach and a fully statistically data-driven view of language processing.

## 3 Basic components and how they aggregate

In this section, we present the notion of sign, which is at the core of our model. First, we detail their representation and second their role in the representation of the meaning under construction, called the *Situation model*.

### 3.1 Representing basic components: the notion of *sign*

**Figure 1:**
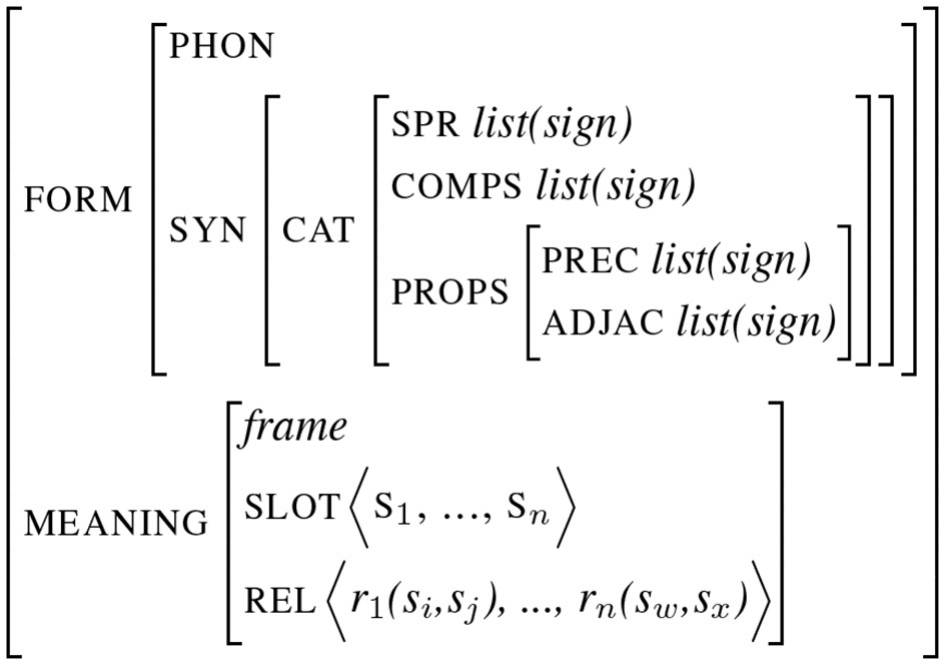
Abstract representation of a sign.

It is now classical to consider linguistic units as complex entities, gathering different types of information. Many linguistic theories such as HPSG or TAG (Joshi and Schabes, 1997; Sag and Wasow, 1999) have developed this perspective by being *lexicalized* in the sense that a lot of syntactic information is already encoded at the lexical level. Pushing one step forward this idea, we have observed how *Construction Grammars* represent linguistic entities as form-meaning pairs: the form part is made of different formal information (phonology, prosody, morphology, and syntax) and the meaning is described by means of semantic slots and relations between them (Fillmore and Baker, 2009). Each sign is then made of different information describing the form and the meaning of the sign itself (corresponding to endogenous features) and its relation with other signs (exogenous features). Figure 1 illustrates an abstract attribute-value representation of a sign, which is adapted from *sign-based construction grammars* (Sag, 2012). The first part of the sign encodes its form by means of phonological and syntactic features, encoding in this example complements and exogenous properties such as linearity or adjacency (the type of constraints proposed in *Property Grammars* proposed in Blache, 2016). This illustration is partial and must be completed with other types of features describing, in particular, phonetics, prosody, or morphology.

**Figure 2:**
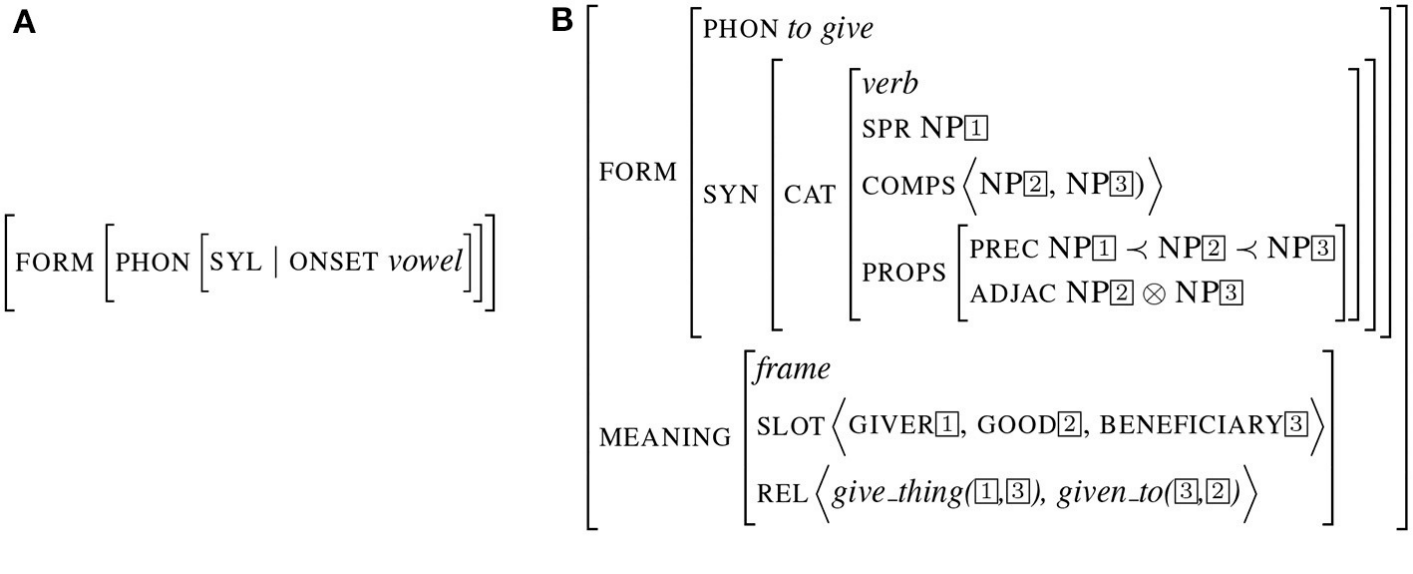
Examples of signs. **(A)** Underspecified sign. **(B)** Predicative sign.

Signs can represent objects at any level. They can be under-specified, restricted to a simple feature as in the example (Figure 2A) representing a word starting with a vowel (as it is the case for words following the determiner “an”). Signs can also encode more complex relations such as a predicative structure as illustrated in Figure 2B. In this representation, the connection between form and meaning is implemented by *structure sharing* (the co-indexation indicates a reference to the same object). In this structure, the same sign (indexed by 1) appears as the value of both the attribute spr (the subject) and the thematic role “giver”. In the same way, the values of the comp (the complements) are shared with different attributes in the meaning description. Note that the meaning not only specifies the slots (in this case, the thematic roles) but also specific semantic relations between slots (implementing a partial conceptual graph).

**Figure 3:**
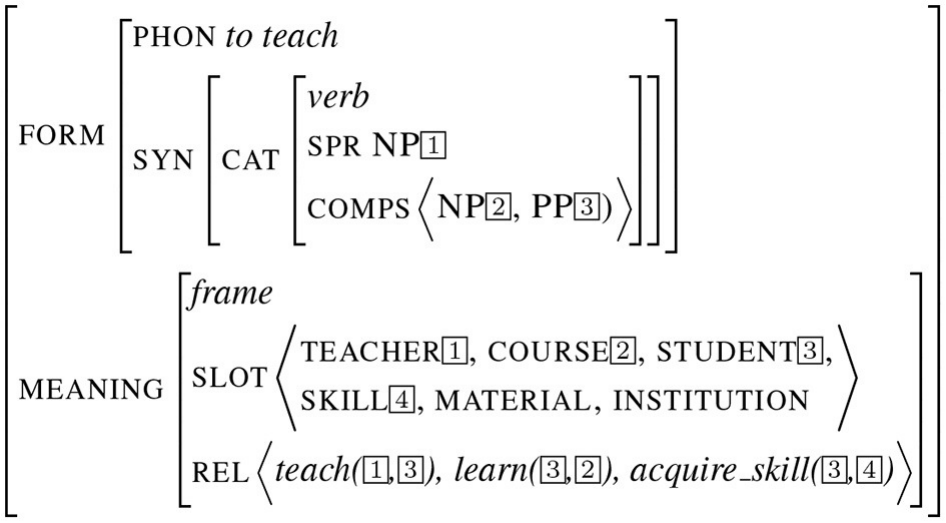
Frame sign.

The third example presented in Figure 3 depicts a sign encoding a more complex piece of meaning, corresponding to an entire frame as described in *Frame Semantics* (Fillmore and Baker, 2009). This example illustrates the frame teaching, bearing a set of slots and relations. Structure sharing implements the form/meaning interface (as it is the case for the subject/teacher values). On the opposite, some attribute values such as material and institution are left free.

#### 3.1.1 Activation, structure sharing

On the one hand, signs encode interaction between different linguistic domains and, on the other hand, their relations with other signs. Structure sharing implements directly relationships between domains, typically between the argument list of a verb (its complements) and the predicative structure at the semantic level.

Signs also encode higher level of information by making reference to other external signs. For example, the predicative structure associated with a verb specifies the type of the arguments that are expected or required. These signs are said to be *activated*, in the sense of ACT-R (Anderson et al., 2004; Lewis and Vasishth, 2005). In this theory, the context provides multiple sources of information that can play the role of cues for a “chunk” (a piece of information), each cue being more or less important in the activation of the chunk (implemented by the weight of the corresponding relation). In our approach, a chunk corresponds to a sign that can be a complex structure with links to other signs. During the processing cycle, the complete context encoded in the situation model is used to identify what information can play the role of a cue for the activation of signs. For example, the verb has a strong relationship with its arguments: instantiating a verb leads to the activation of its arguments, making them available for future instantiation.

#### 3.1.2 Are signs testable?

All linguistic theories have shown the necessity to consider the basic components of language processing as complex objects such as signs. These theories have shown more precisely that the notion of linguistic category has to integrate many different types of information from many different domains. In other words, the type of information associated with words is necessarily complex. Any language processing model has to take this question into consideration. When addressing the question of experimentation, this becomes of course an issue: are such complex objects testable in a neuro-linguistic perspective? Different works in neuroscience use complex signs as basic component: we have observed the form of lexical entries in MUC, but other works have also shown the encoding in the brain of more complex constructions in terms of neuronal assemblies (Pulvermüller et al., 2013). At the experimental level, testing signs in their complete complexity is of course difficult. However, it is possible to test one of their subparts by controlling the rest. For example, when studying semantic or syntactic violations, the tested subpart is one of the features of the structure. Moreover, even though signs are a very powerful representation, they can and should be used in experiment when we are trying to explain language processing in general (and not one of its subparts).

### 3.2 The *situation model* as a graph

**Figure 4:**
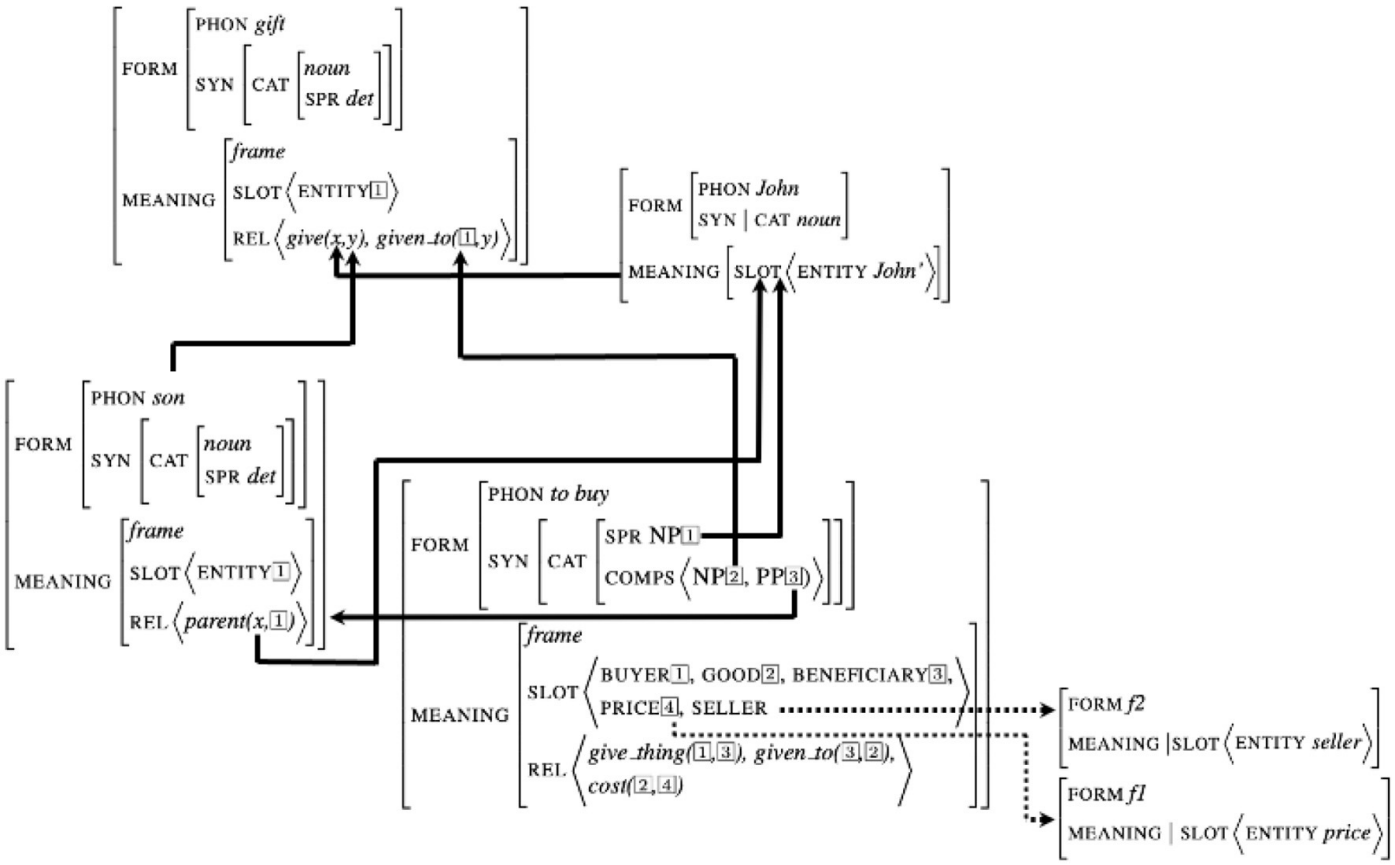
Graph implementing the situation model of the sentence “John buys a gift for his son.”

Theories of interaction describe understanding as the possibility for each participant to build a “*situation model*”, consisting in the set of implicit and explicit knowledge conveyed during a conversation (Zwaan and Radvansky, 1998; Pickering and Garrod, 2021). In our proposal, such a knowledge is built upon the set of signs instantiated during the conversation. Any sign can bear a set of relations with other signs, implementing syntactic and semantic dependencies. The representation of this knowledge consists therefore in a graph in which nodes are the signs and edges represent dependencies. The example in Figure 4 presents such a graph encoding the meaning of the sentence “John buys a gift for his son”. This graph is based on the predicative structure associated with the verb “to buy” connected with its arguments John, gift, and son. Note first that due to the structure of the signs, dependencies are implemented within a sign by structure sharing. The relations connect the internal arguments of a sign (its slots) with external signs, leading to the unification of both structures. For example, the subject of the verb to buy (in this sign-based representation encoded by the specifier attribute spr) is connected with the sign John, which to its turn is connected with the first argument of the semantic relation *give* of the noun gift.

Note that the conjunction of unification between internal slots and external signs together with structure sharing within signs ensures the propagation of all dependencies inside and outside the structures. For example, when the values of the attributes spr and comps of the sign to buy are unified with the lexical signs John, gift and son, this information is propagated within the sign to buy to the different arguments co-indexed in the structure: John and gift become then the arguments of the relation *give_thing*.

The example in Figure 4 illustrates another property that is implicit in knowledge graphs: *activation*. This type of relation, represented in the figure by dotted lines, connect slots with sign not already realized in the discourse. For example, the semantic structure of the verb to buy contains the slot price and seller that are not instantiated in the sentence. They are considered to be *activated* which means that this information become available for future unification.

## 4 Unification and the updating of the *situation model*

We present in this section in more details unification mechanism and how it is involved in the situation model updating.

### 4.1 Unification

Unification is a mathematical operation consisting in comparing two structures and assessing their compatibility (Robinson, 1965). This mechanism is at the basis of logic programming (Colmerauer, 1986) and most current linguistic theories (Shieber, 1986). As presented above, this notion is also at the core of the neuro-linguistic model MUC, considering unification as the main mechanism implementing the combinatorial nature of language (Hagoort, 2013). Technically, unification is implemented as the resolution of an equation system, the solution being a substitution making the two-term structures identical.

The first important characteristic of unification is that any type of structure can be compared. In the simplest case, the two structures are fully specified, and unification simply consists in verifying that these structures are identical. If this happens, unification succeeds, otherwise it fails. For example, the predicates f1=buy(John,gift) and f2=buy(John,bike) are not identical and their unification (noted f1 ⊔ f2) fails.

However, the most interesting case occurs when two underspecified structures are unified. In the case where one of these structures has an argument with a free value (a variable), unification consists in finding a substitution between this variable and the corresponding argument in the second structure to be unified. For example, unifying buy(John,gift) with buy(John,x) succeeds on condition that x=gift. Unification then leads to instantiation of the free variable x in the resulting unified structure. Of course, substitution can be applied to two free variables: unifying buy(John,x) with buy(John,y) succeeds on condition that x=y. This means that both variables x and y remain free but with the constraint to be equal in the case of future instantiation.

**Figure 5:**
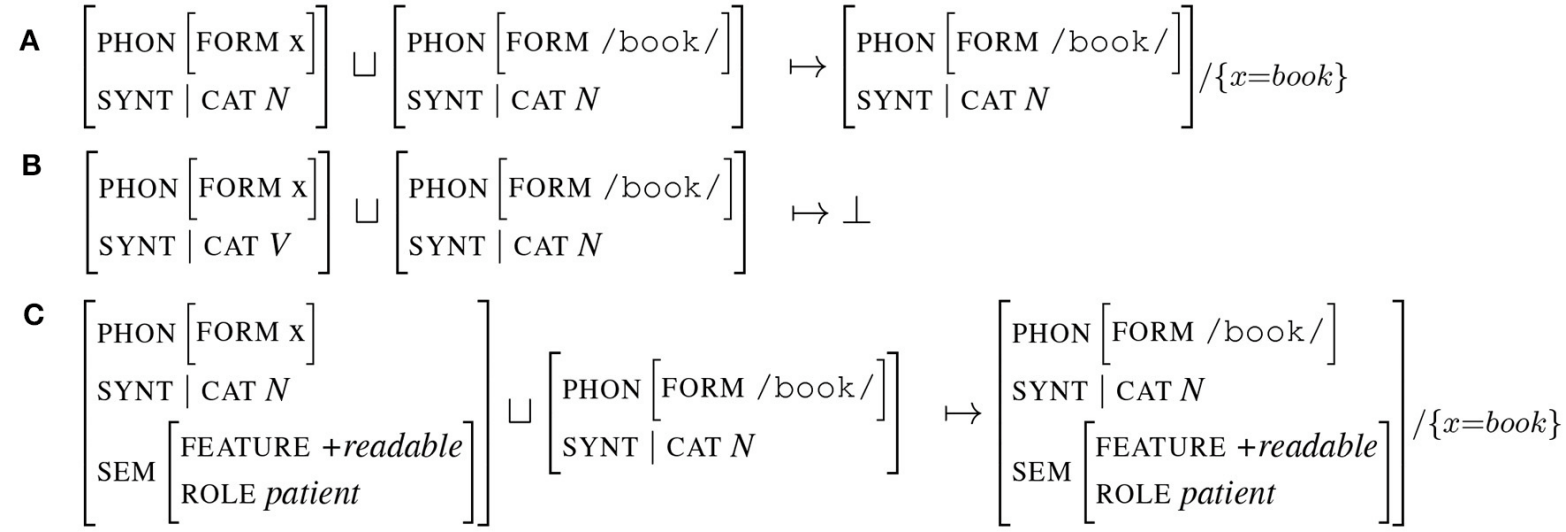
Signs unification.

Unification applies to any type of structure and can therefore be applied to entire signs.^2^ In this case, unification consists in comparing term by term the attributes of the signs to be unified. The example in Figure 5A illustrates the case where two signs representing a noun (one having its form instantiated the other not) unify. The result is the third sign, which is associated with the substitution x=book. This substitution information will be propagated to all other instances of x. The example Figure 5B illustrates an unification failure: the attributes cat of the unified signs are not compatible, one being *V*, the other *N*. Finally, example Figure 5C illustrates an important property of unification: instantiation. In the case where one sign contains an attribute which is not specified in the other sign, unification succeeds and the resulting sign is the union of the arguments of both signs. In example Figure 5C, the attribute sem is only instantiated in the first sign. As all other attributes are compatible (equals or by substitution x=book), the resulting unified sign is the fusion of both signs. We will observe in the next section the importance of this mechanism when unifying predicted and produced signs. Overall, as summarized in the study by Huettig et al. (2022), the result of unification is a sign that shares all the features they have in common and preserves all the features that are distinct.

### 4.2 Unification and the brain

In the MUC model, the basic information components are retrieved from the long-term memory and assembled by unification into larger structures in order to derive high-level meaning. The comprehension process during a conversation is considered to be sequential. The different linguistic components (words, chunks, expressions, etc.) are therefore integrated incrementally in two steps: the information corresponding to the component is first retrieved from the lexicon and then integrated into the structure under construction thanks to unification.

In MUC, the distinction between memory and unification is found at the cerebral level. Temporal and parietal regions are crucial for memory retrieval. For its part, the left inferior frontal gyrus is recruited in unification operations at different levels (Hagoort, 2005). Lexical processing involving unification into representations spanning larger multi-word structures activates different areas in this brain region. A distinction is done between different types of unification that can also be associated with this region (Hagoort, 2013): semantic unification (Brodmann's areas BA47 and BA45), syntactic unification (BA45 and BA44), and phonological processes (BA44 and ventral parts of BA6). Different experiments have shown how these regions can be differently activated, reflecting the unification load, for example, when processing semantic incongruities (Hagoort, 2016).

### 4.3 The updating mechanism

*Situation Model* instantiation is based on unification. Signs encode in a unique structure lexical, syntactic, and semantic information, which is processed at the same time. This is an important feature of our model; unlike other approaches such as MUC, we do not distinguish between syntactic and semantic unification, and more importantly, there is no chronology between them. The question is then to explain how the different signs can be aggregated during the processing. Taking the hypothesis that language processing is incremental, achieved token by token, a new sign has to be interpreted at each step and integrated into the structure under construction (the situation model). This integration is done by unifying information which is expected with the one associated with the input sign.

#### 4.3.1 Activated sign

The situation model is a graph of signs, each sign being formed with features (or attributes) and values possibly connected with other signs (the value of a feature being possibly a sign). For example, the value of the feature spr in Figure 2B is a sign corresponding to an *NP*. This representation means that the sign describing the verb to give expects *NP* as subject. The situation model is then made of a graph of two types of signs: (1) signs that have been *realized* in the input signal (a word produced by the speaker) and (2) activated signs that are not already realized (feature values of type *sign*). Activated signs are underspecified, bearing partial characteristics of the type of information that should occur at this position.

For example, in Figure 4, the signs corresponding to the slots price and seller encode information not realized in the discourse. Activated signs bear a partial semantic information, with no specification about the form. They encode information available for the integration of possible future realized signs.

Remind that activated signs can be activated either by semantic or syntactic relations, with no distinction: both forms and meaning features can potentially have attributes with values of type sign. This is, for example, the case of the the syntactic features comps, specifying the complements. In Figure 4, when encountering the word buys, the signs corresponding to the *NP* and *PP* complements become activated signs, which are available for future integration.

#### 4.3.2 Weighted activation

Situation model encodes different expectations. However, not all of them are at the same level: some can correspond to important or even compulsory information, some others being more optional. All relations in the graph bear a weight encoding its importance: mandatory complements bear high weights, optional signs a light one. This information is fundamental in the estimation of the *activation level* of a sign, in the sense proposed in ACT-R adapted to language (Lewis and Vasishth, 2005). In this approach, activation is evaluated based on different information. Each sign in the situation model has a *basic activation*, based on the frequency and the history of its access. This value is completed with the sum of the weights of the different cues in relation with the sign. Concretely, in our graph-based representation of the situation model in which each node corresponds to a sign, the more a sign receives input relations, the higher its activation. Different approaches for estimating the activation level have been proposed, completing the ACT-R proposal. For example, Property Grammars encode all linguistic relations by means of constraints (Blache, 2013). The state of the constraint system associated with each sign is analyzed, comparing, in particular, the sum of the weights of satisfied constraints with those unsatisfied. This indication is then integrated to the activation level estimation. Note that at the brain level, it has been observed that the amplitude of the N400 evoked by a word is negatively correlated with the activation level (Baggio and Hagoort, 2011).

#### 4.3.3 Sign integration

Integrating a new sign into the situation model graph consists therefore in unifying it with an expected sign. More precisely, the situation model contains a list of activated signs with different activation values. The integration of the new sign into the graph is implemented by looking in priority for its unification with highly activated signs. Note that the activation level, as proposed in ACT-R, can increase with the number of relations that the activated sign receives. For example, the sign *John* in Figure 4 receives directly two input relations (plus indirect ones due to structure sharing within the sign *buy*). As a consequence, it becomes the highest activated sign in the situation model and thus used in priority for future integration.

#### 4.3.4 Unification mismatch and loose unification

In addition to the estimation of the activation level, feature weights also play an important role in controlling unification. Weights, by distinguishing between important and optional relations, offer the possibility to implement unification as a flexible mechanism. We have observed that unification fails when two feature values are not compatible. If the mismatch involves features with heavy weights, unification failure is hard, which is not recoverable. On the opposite, when incompatible features have light weights, it is possible to consider that unification succeeds anyway by relaxing the equality constraint for these specific values.

Unification of two complex signs provides a way to precisely identify (and quantify) for what reason unification succeeds or fails, which is of deep importance in the study of different types of mismatches. As a consequence, weights offer the possibility to evaluate the strength of unification success or failure, giving a way to estimate a gradient between light and hard failures. Interestingly, this gradient could be correlated with the nature and the amplitude of the response in the brain signal faced with incongruities. The result of the unification between two signs with a large amount of compatible heavy features will be considered as more complete, stable, and saturated than a resulting sign requiring constraint relaxation.

In addition to weights, semantic similarity constitutes a second information that can be used for identifying situations of mismatch allowing loose unification. When the expected sign and the sign produced by the speaker do not unify, a semantic distance can be calculated in the same way as distributional semantic does (distance between embeddings). If this distance is low, failure can be relaxed and unification succeeds. This type of phenomenon has been observed at the brain level: the amplitude of the evoked potential N400 is proportional to the semantic distance (Baggio and Hagoort, 2011; Nieuwland, 2019).

#### 4.3.5 Disconnected graph for representing the semantic structure

The semantic knowledge can be made of different part of information, not necessarily connected together. Encoding the situation model as a graph offers this important property: it can be disconnected. During a conversation, it can be the case that at some point, a new information is introduced with no explicit relation with the previous context. In such case, this new information is buffered as a disconnected sub-graph that can be connected later on during another updating of the situation model.

## 5 Prediction

Language processing is based on prediction: inferences using high-level information can be done in order to predict what will happen at lower levels (Pickering and Garrod, 2007; Kuperberg and Jaeger, 2016; Heilbron et al., 2022; Ryskin and Nieuwland, 2023). During a conversation, the listener anticipates different types of information before encountering the input produced by the speaker. By doing this, participants use the context to facilitate processing. Many studies in psycholinguistics have underlined this role of prediction (Ferreira and Chantavarin, 2018). Highly predicted words are read more rapidly (Monsalve et al., 2012) and elicit facilitation patterns in the brain signal (Kutas and Hillyard, 1984; Hagoort et al., 2009). We describe in this section the main features of prediction and its role in our model as a facilitation mechanism.

### 5.1 What is predicted?

It is important to precise what exactly is predicted under what form and at what level. First, let us recall that language processing is classically considered to be incremental and processed word-by-word. The mechanism consists in updating hypotheses after encountering each incoming word (Kuperberg and Jaeger, 2016). In most models (in the same way as with deep language models), prediction concerns the next word. However, several studies have shown that predictions can be done over multiple levels of representations (Caucheteux et al., 2023). For example, one can predict a phonetic constraint based on the previous word (DeLong et al., 2005): the use of the determiner /a/ (resp. /an/) stipulates that the next word should start with a consonant (resp. a vowel). In the same way, at the lexical semantic level, the use of a certain predicate (e.g., the verb “to drink”) may impose a semantic feature on the argument (e.g., [+drinkable]). Predictions can capture coarse or fine-grained properties (Wang et al., 2020; Ryskin and Nieuwland, 2023) at any level: morpho-syntactic category, semantic type, phonetic structure, and complete events or frames (Huettig, 2015). Predicting representations with such a multiple granularity requires to take into account the complete narrative context, the participant's world knowledge, theory of mind, etc.

As a consequence, when related to the predictive coding hierarchical architecture, this means that prediction can be done at any level of representation. It can also be done at any moment: whatever its granularity, being it fine or coarse, using the entire context available at a time *t* makes it possible to predict either a single feature (in the case of poor contextual information) or on the opposite a complex structure, bearing a rich bunch of information. This means that prediction is not necessarily done at a unique level, step-by-step, going linearly from one level to another but can be done in a single shot, simultaneously at any level. We will observe that this sequential, incremental, and hierarchical architecture is a general theoretical proposal, possibly shortcut by shunting some levels due to the context.

**Figure 6:**
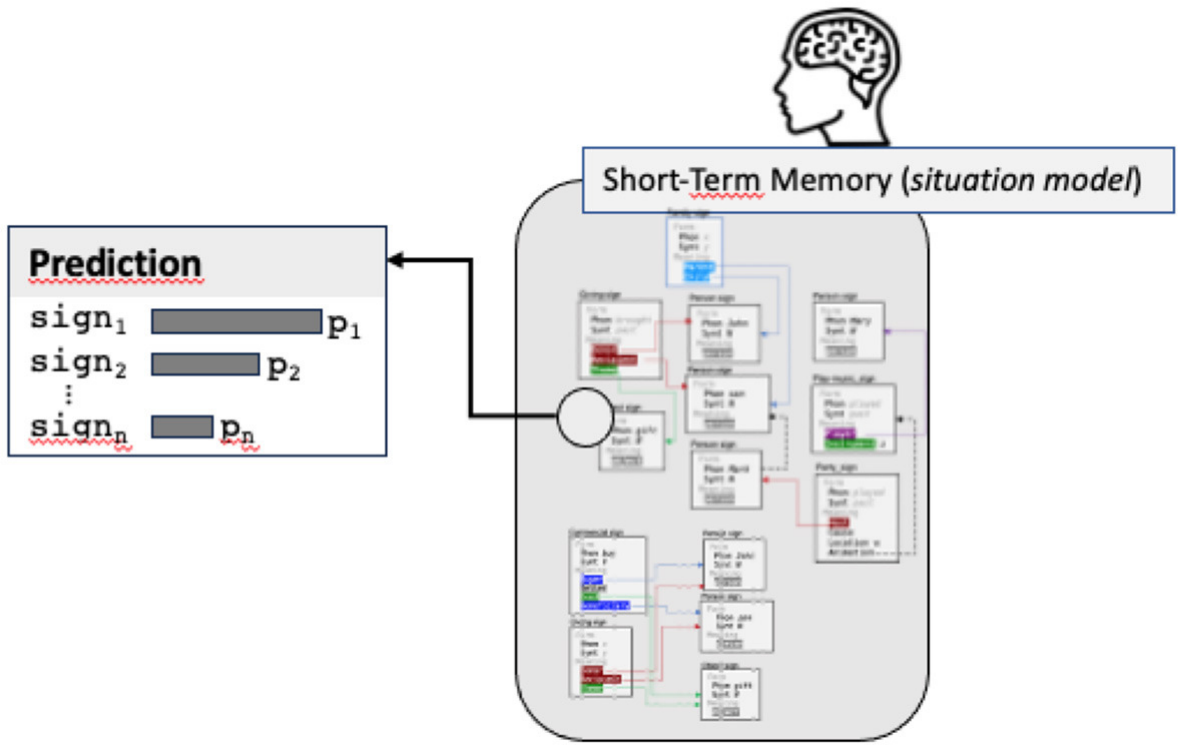
The prediction step returns a list of possible signs, calculated from the situation model, with their probabilities (the image of short-term memory is simply an illustration of the situation model graph, no need to read the content of the nodes).

In addition to granularity, the second important feature of prediction is that it is a probabilistic phenomenon: multiple predictions can be done in parallel (for example corresponding to multiple parses). There is in this case a *graded pre-activation of multiple candidates within long-term memory* (Kuperberg and Jaeger, 2016; Frisson et al., 2017). As a consequence, the prediction process, instead of providing a single output, returns a list of predictions with their probability, making it possible to order them from high to low predictions (see Figure 6).

Note that each predicted sign is lexically anchored in the sense that it contains necessarily a prediction about the expected surface form, starting with the next token. Such prediction is not necessarily the exact phonetic form and can be some formal properties about the form. For example, the predicted sign (including when it concerns an entire frame) can stipulate a phonological constraint (e.g., that the next token should start with a vowel), a morphosyntactic category (e.g., the next token should be a noun), or even a lexical form. In other words, prediction can concern signs at any level (a word, a construction, or a complete semantic frame) but always stipulate constraints on the next token to be produced by the speaker.

### 5.2 Predictive coding

The *predictive coding* paradigm in neuroscience is based on the fact that the brain is an inference organ, constantly making predictions about events that are going to happen depending on the context (Rao and Ballard, 1999; Friston, 2018; Pezzulo et al., 2021). During perception, the brain makes predictions at each stage of processing and compares these predictions (coming from the brain) with the perceived signal (coming from the senses). Each comparison measures a distance between the two signals, resulting in a *prediction error*. Therefore, there is a constant double movement, bottom-up and top-down, during which each step provides a measure of the prediction error that propagates in both directions. This paradigm relies on a hierarchical architecture from lower levels (sensory inputs) to higher levels, encoding abstract information. When applied to language (see Ryskin and Nieuwland, 2023 for a review), the classical hierarchical structure of processing (from sound to sense) represents this sequential architecture in which each level represents a type of linguistic processing: lower levels of the hierarchy correspond to sounds, the higher level is the meaning, intermediate levels are syllables, words, phrases, constructions, syntactic structures, etc. In this strict hierarchical organization, the predictive processing consists for high-level predictions to inform low-level ones: top-down predictions from higher levels are at each step compared with perceived stimuli.

The question of prediction is of course topical in language processing. Artificial intelligence and large language models are entirely based on predicting what comes next. During conversations, this is done by activating different sources of information from word to world knowledge. Concretely, in a comparable manner as for natural language processing, predictions are generated by models based on prior experience with the event knowledge. Each level in the hierarchy corresponds to a model making a prediction based on the context (the context being what has been realized and interpreted). These generative models provide top-down predictive signals from hierarchically higher levels which are compared with incoming stimuli. The prediction error (i.e., the mismatch between the prediction and the sensory signal) is propagated to higher layers in the hierarchy. In other words, prediction is transmitted down from higher levels to the lower levels, whereas prediction error is propagated upward. This mechanism makes it possible at each step to update the response and generate the next prediction by revising the weights of the hypotheses. A minimization of the prediction error is then applied progressively, improving each model predictions, until reaching the best interpretation of the input signal.

When studying the brain correlates with this architecture, where each layer predicts a neural activity of the level below, the evaluation of the prediction error consists in comparing the predicted activity with that of the current layer. This architecture explains how responses are modulated by linguistic predictions (Heilbron et al., 2022). Many types of prediction mismatch have been explored in the literature. Typically, an incongruous word involves a large prediction error generating a strong negative component, the N400 effect (Kutas and Hillyard, 1984). Earlier components related to prediction have also been observed, for example, the ELAN effect associated with syntactic errors (Friederici et al., 1993) and positive effects (see Nieuwland, 2019 for a review).

Predictive coding perfectly fits with what is observed in language processing, including through different experiments in NLP looking for a mapping between the activation of artificial neural networks and brain areas (Caucheteux et al., 2023). This notion of top-down prediction applied step-by-step in a linear way is very productive and fits well with the classical hierarchical organization of language processing going step-by-step through the different linguistic dimensions: phonetics, phonology, morphology, syntax, and semantics. However, as observed in the previous section, the integration mechanism can be non-linear in the sense that what is predicted at each processing cycle can be at very different levels: from low-level underspecified signs until richly instantiated signs encoding a large piece of meaning. At the beginning of a processing cycle, when few information is predicted, the mechanism is basically linear. On the opposite, when whole pieces of information are predicted, the different layers are skipped and the processing cycle goes directly to the integration step. This non-linearity corresponds to the main form of facilitation. Note that, as we will observe it in the last section, non-linearity is also at work within a processing cycle during stage change: the activation, saturation, and probability level of a sign decides for activating of inhibiting specific mechanisms such as lexical access.

### 5.3 Prediction vs. activation

In our model, we distinguish between **prediction** and **activation**. Following the predictive coding framework, we consider that prediction is always at work in language processing. However, the mechanism is different depending on the scope of the expectation. In their review paper, Van Petten and Luka (2012) propose to distinguish between *prediction*, the mechanism explicitly devoted to the anticipation of a specific word to occur in the future), and *expectation*, which refers to the broad semantic content that can be anticipated. In our model, we use a comparable distinction between *prediction* and *activation*:

- *Prediction*: The mechanism that calculates the most likely next sign based on the context.
- *Activation*: The mechanism that calculates all activated signs, not already realized, regardless of whether they will be realized or not.

The notion of *prediction* adopted here is local in the sense that it is restricted to the next event, predicted word-by-word. It refers to the pre-activation of a linguistic representation before verification with the bottom-up input produced by the speaker. For its part, the notion of *activation* is global and refers to the meaning encoded in the complete situation model. At each processing cycle, the situation model is updated first by aggregating the new sign corresponding to the word produced by the speaker and second by adding to the model all the signs activated by enough cues. As a consequence, the situation model encodes two types of information: (1) a set of signs connected by relations, corresponding to what was actually produced by the speakers and (2) expectations about what might be produced during the rest of the conversation. Such expectations correspond to *activated signs*, not already realized and representing a possible slot value. For example, when encountering the verb buy in the sentence “John buys a gift to his son”, two signs corresponding to the complements (*NP* and *PP*) are activated (see Figure 4). Activation, unlike prediction, says nothing about the position or the moment when the activated sign could be realized. It simply prepares a future possible unification.

On the other hand, it is possible to predict step-by-step, at any time, the next word (or more precisely a list of possible following words) to be produced. Large language models have shown the effectiveness of this mechanism. If we simply extend the notion of word to that of sign, we can make the hypothesis that at each processing cycle (i.e., each incoming word), a new sign (more or less specified depending on the context) can be predicted.

To sum up, **activation** specifies the set of signs corresponding to expected pre-activated values, while **prediction** is restricted to the next sign to be realized. Activation defines a set of activated signs associated with an activation level depending on the context (see Section 4.3). Prediction specifies at each position *t* in the input signal a set of signs that can appear at *t*+1, ranked by probability.

## 6 The processing cycle of the prediction-unification model

Prediction and unification cannot be separated in this type of model. Unification represents the unique mechanism for comparing the predicted structure with the produced one. Moreover, it is a constructive process making it possible to build a new structure by merging the unified ones. Involving prediction into the model requires unification. We will describe in this section the role played by unification at each step of the processing cycle.

We propose in this section to present the processing cycle by taking the case of a conversation, even though the same cycle can be applied in reading, by substituting the audio input to a written one.

### 6.1 Segmenting

The first step consists in segmenting the input.^3^ Different studies have explored for many years how infants and adults can segment the input audio stream and identify syllables, word, or phrases boundaries (Saffran et al., 1996; Mattys et al., 1999; Endress and Hauser, 2010; Matzinger et al., 2021). Several different cues are used in this task: prosodic (lengthening, pause, and pitch change), phonotactic (permissible combinations of phonemes), statistical (transitional probability between adjacent syllable), etc. Note that automatic speech recognition techniques also rely on similar cues (Georgescu et al., 2021) and can be correlated at the brain level (Lee and Cho, 2016).

These studies show that speech segmentation can be based on low-level features, explaining that this skill appears early in language development and is extremely efficient. When studying the complete cycle of language processing during natural interaction, segmentation provides the first input information: this preliminary step returns a sequence of phonemes (or at least a set of acoustic features in noisy environments). Remind that in our architecture, the basic linguistic units are constructions, which are made of a form/meaning pair. In this framework, all types of linguistic objects (including words) are considered as constructions and represented with this same format. As a consequence, the phoneme sequence returned by the segmentation mechanism can be used as a key for accessing the mental lexicon by comparing the sequence with the form attribute of the corresponding word in the lexicon. The important characteristics are that this segmentation is done early and of course way before accessing any information related to the word.

In classical language processing architectures (as well as in linguistic theories in general), this segmentation is usually associated with lexical access, in a unique step: segmenting a word consists in retrieving in the lexicon a matching sequence of phonemes. The information associated with the word (its morpho-syntactic features and its meaning) is systematically retrieved from the mental lexicon, for all words. We propose in our model to clearly distinguish between segmentation and lexical access. By considering segmentation as an independent process, we make it possible to use these segments in different purposes. They can be used as a control mechanism for verifying whether what is predicted by the hearer corresponds effectively to what has been produced by the speaker. They can also serve as a key for retrieving the corresponding word stored in the mental lexicon.

**Figure 7:**
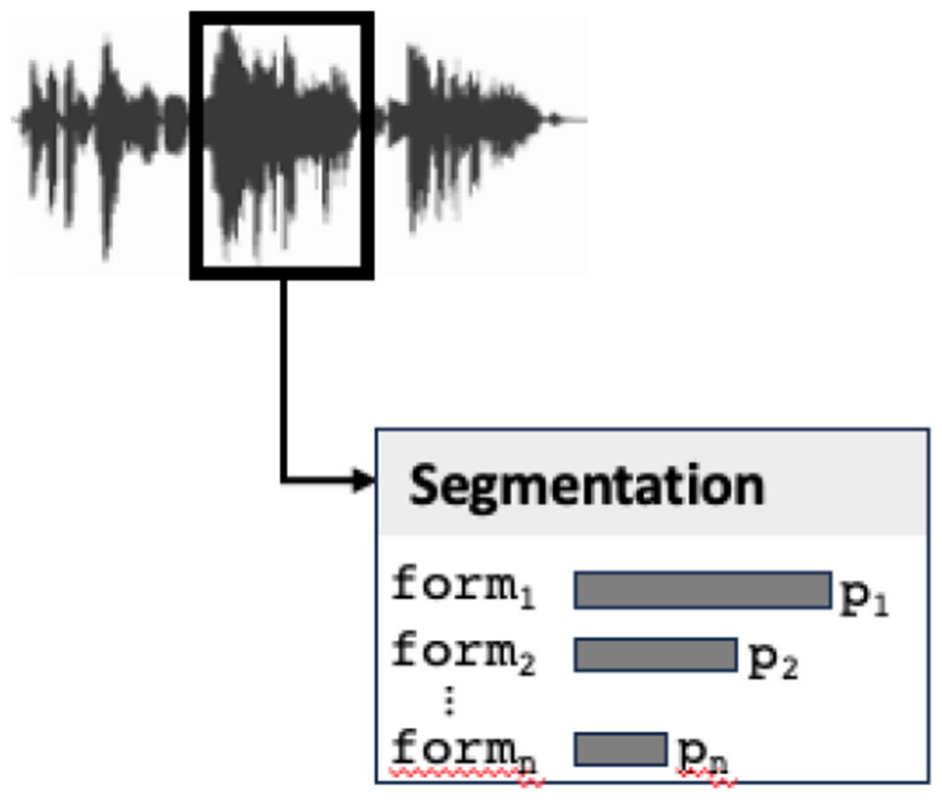
The segmenting step returns a list of possible forms (audio segments) with their probabilities.

As observed above, audio stream segmentation returns a set of phonemes, possibly incomplete in the case of noisy signal (see Figure 7). This step can of course be prone to errors or uncertainty. Each element of the sequence, instead of being a phoneme, is rather a probabilistic space representing possible phonemes. As a consequence, the segmenting step of the audio input calculates a list of possible audio segments. In terms of a sign-based representation, an audio segment corresponds to a value of the phon attribute in the form description. Segmenting returns therefore a list of signs specified only for their form value, each sign corresponding to a possible audio segment (with its probability).

### 6.2 Comparing the predicted sign with the input audio segment

Predictive coding is based on the comparison between top-down prediction and bottom-up information. In most models, this comparison is done at each level of the processing in a hierarchical sequential way (Ryskin and Nieuwland, 2023). In the case of our model, we integrate the fact that complex signs can be predicted. The first step consists therefore in comparing what has been produced by the speaker (the audio segment, corresponding to a word) with the form of the predicted sign. Remind that prediction and segmentation steps return a list of possible signs, more or less specified, and ranked by probability. The comparison between the predicted sign and the input stimulus consists in verifying by unification the compatibility of both signs. Concretely, this consists in unifying a partial sign returned by segmentation (containing only the form attribute) with the form value of the predicted sign (or with the beginning of the form value when multiple words are predicted).

**Figure 8:**
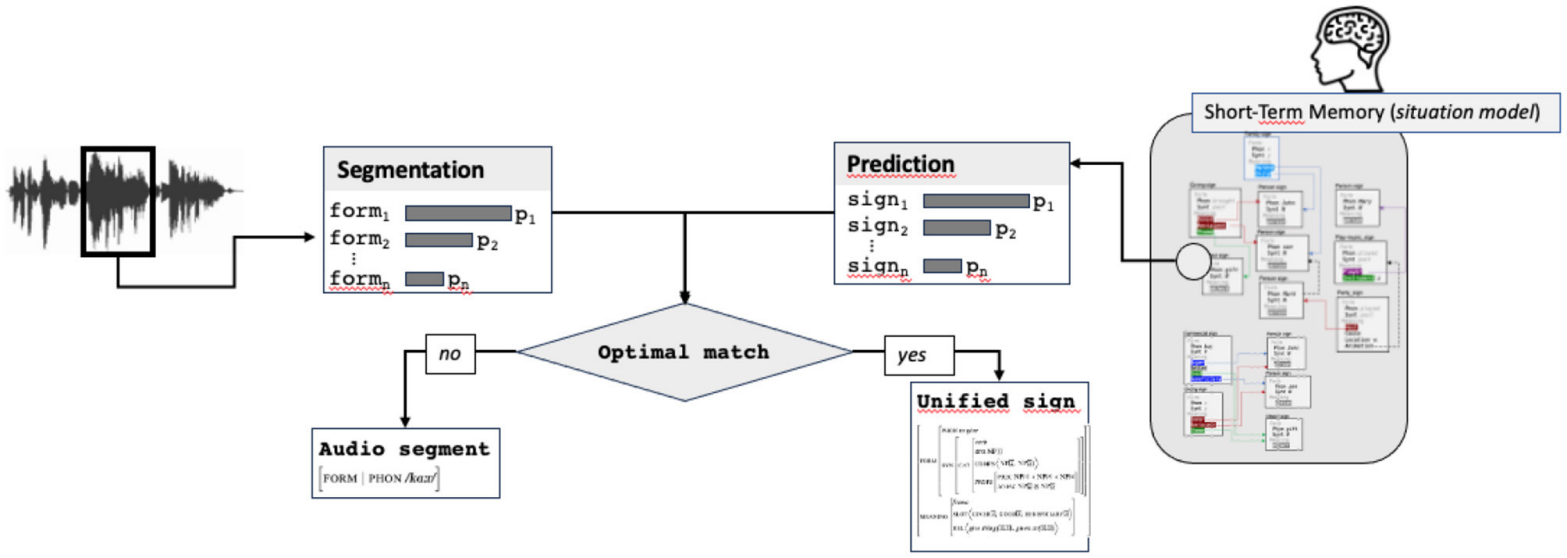
Comparison stimulus/prediction, returning the optimal match, possibly reduced to the audio form only.

If unification succeeds, the sign resulting from the unification (called in Figure 8 the “*Unified sign*”) is passed to the next step. This sign contains all predicted information plus the audio form and can correspond already this stage to a more or less complete sign, containing a lot of information. If unification fails, a new match is sought by finding the next more likely pair audio segment/predicted sign. If no match between any audio segment and predicted sign exists, the most probable audio segment, forming an underspecified sign containing only the form attribute, is passed to the next step.

### 6.3 To access the lexicon or not: prediction and saturation

When prediction matches with the input stimulus, the resulting unified sign contains, on the one hand, the phonetic form coming from the speaker's production and, on the other hand, all predicted information coming from hearer's prediction. It is necessary at this stage to estimate whether the unified sign can be considered as complete before updating the situation model.

Depending on the state of the context encoded in the situation model, this predicted sign can be more or less instantiated or specified. Remind that a sign is made of a set of weighted attributes, some of them being mandatory. It is thus possible to estimate what we can call a *saturation level*. This notion is borrowed from HPSG (Sag and Wasow, 1999) where a sign is said to be saturated when all mandatory complements are realized. We extend this notion by introducing a gradient making it possible to estimate a level of saturation. The saturation level is calculated as a function of the attribute weights, in the manner of Blache (2016): a high number of attributes with heavy weights results in a high saturation level.

The second important input for estimating whether the unified sign contains information enough for being used directly is the **probability level** of the predicted sign. Intuitively, the idea is that the probability level of the predicted sign gives also an indication on its completeness. When the probability of the predicted sign and the saturation level of the unified sign are both high, it is very likely that it contains information enough and can be directly used for updating the situation model, without accessing the lexicon. On the contrary, when these levels are low, it indicates that the unified sign does not contain information enough. In this case, the lexicon is accessed, looking for a corresponding entry.

The necessity to access the lexicon also occurs when there is no possible matching (even by relaxing some constraints) between the audio segment and the predicted sign. In this case, priority is given to the stimulus, and a matching lexical entry is searched in the lexicon, based on the phonetic form only.

**Figure 9:**
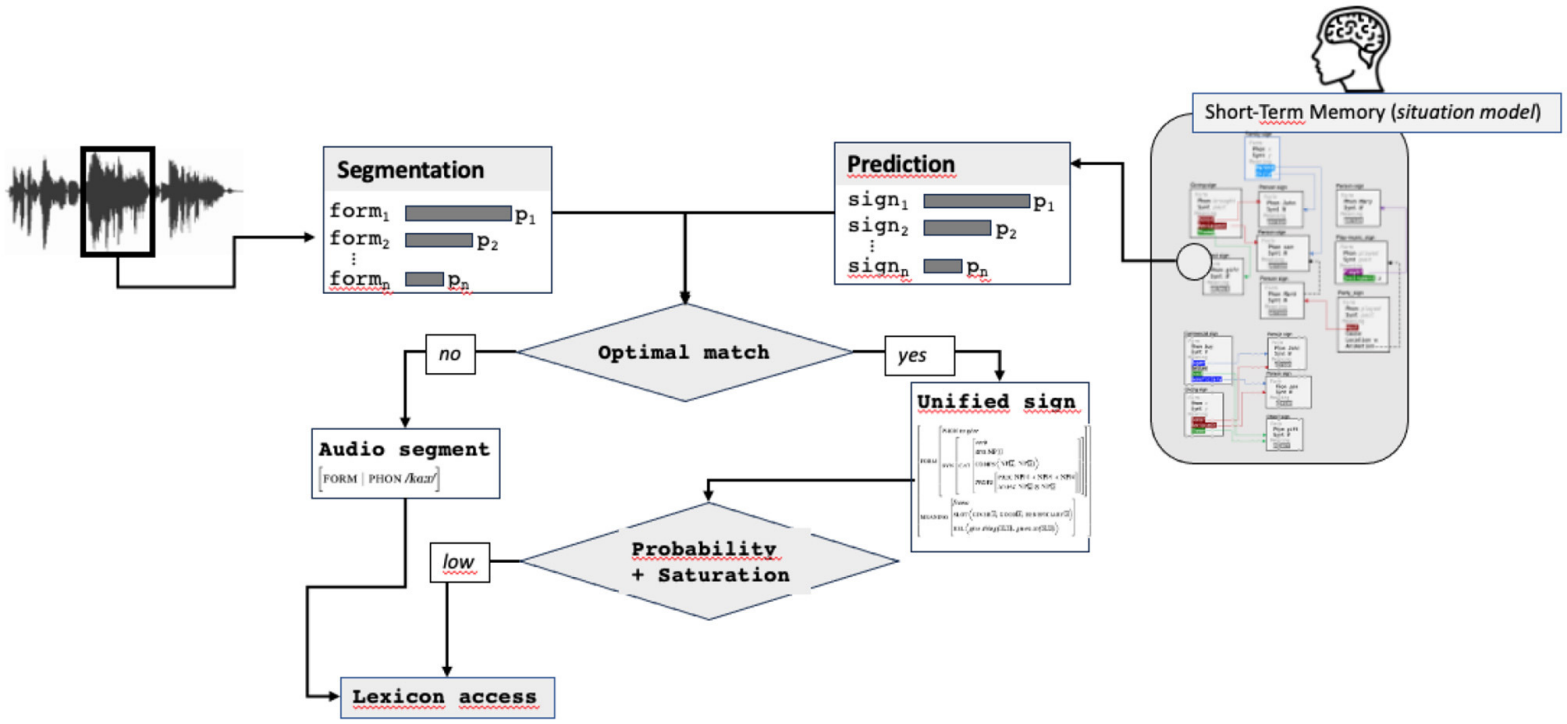
Estimation of the saturation level, access to the lexicon.

To sum up, accessing the lexicon is thus required when the unified sign bears not enough information or when a mismatch occurs between the audio input and the predicted sign (see Figure 9). Technically, lexical access consists in looking for an entry in the lexicon unifying with the sign resulting from the first step. This sign constitutes the *access key* to the lexical database formed by the mental lexicon. Note that the higher the access key specification, the more efficient the access: when the the access key contains a lot of information, the search space in the lexicon is reduced, each information playing the role of a constraint pruning the lexical space. As a consequence, accessing the lexicon when the only available information is the phonetic form (i.e., when there is no match between audio segment and prediction) will be more demanding than when some predicted information can be used. Concretely, accessing the lexicon consists in unifying the access key that can contain predicted information and the lexical entry. The result of this unification is a *retrieved sign* that will be passed to the next step.

**Figure 10:**
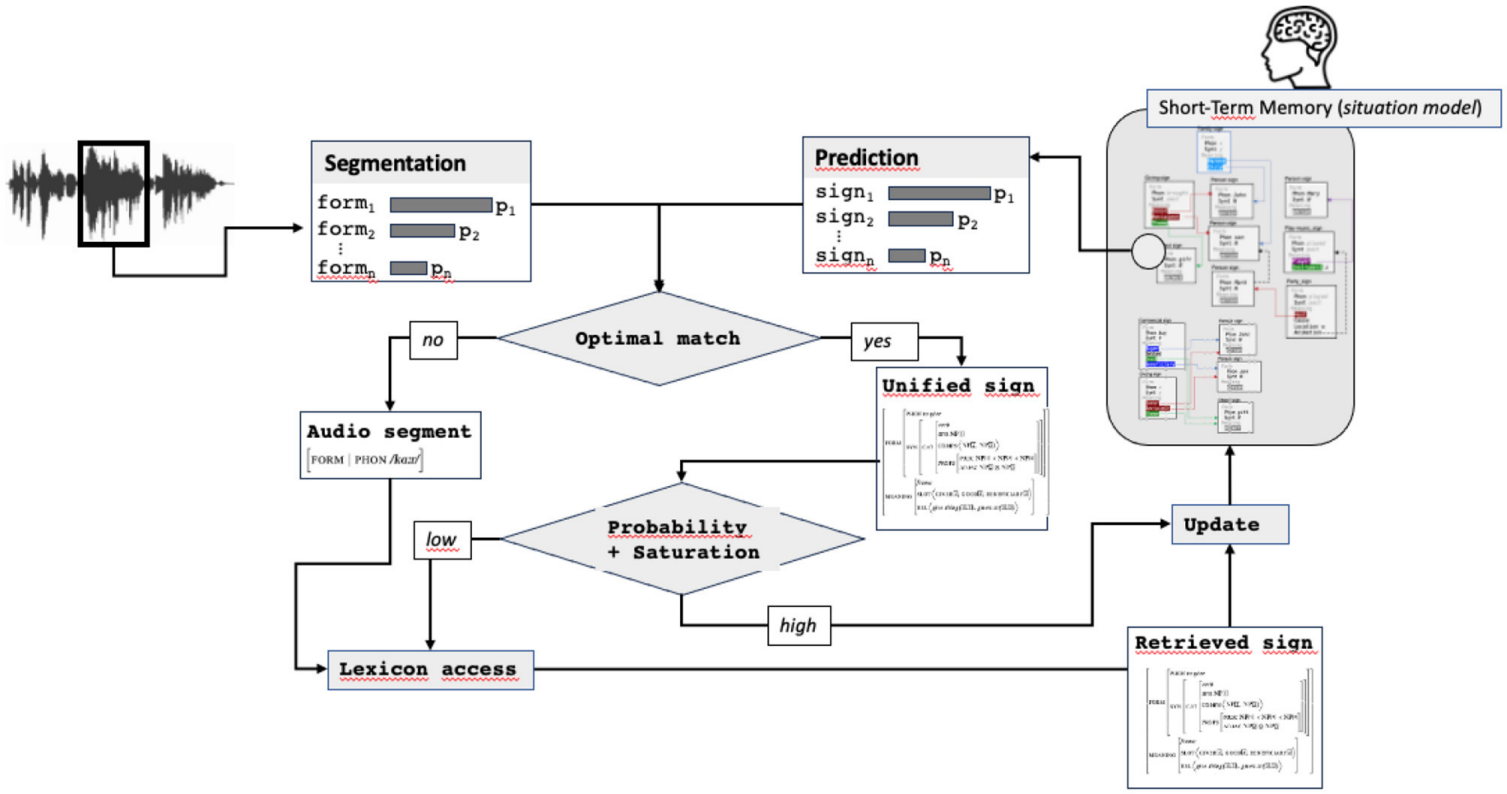
The complete prediction-unification model.

Two routes can be taken at this stage in the processing cycle: a facilitation route and a normal one. The facilitated route corresponds to the situation where the predicted sign matches with the audio segment and contains information enough to be considered as saturated and probable. In this case, the sign resulting from the prediction and the audio segmentation (called *unified sign* in Figure 10) is directly passed to the updating process, without needing any lexical access, which constitutes the first main facilitation mechanism.

The second route involves lexical access. It is used in two cases: when no match can be find between an audio segment and a predicted sign or when a match exists, but the unified sign does not contain information enough. In this case, the lexical access, completed with the audio form and predicted information, results in a sign (called *retrieved sign* in Figure 10) used for updating.

### 6.4 The complete model

The previous steps in the model result in building the sign that will be used in the final updating step of the cycle: the integration of the sign to the situation model. Integration consists in scanning the situation model, looking for an activated slot unifiable with the updating sign.

This model makes it possible to take into account the two types of mechanisms at play in language processing : the normal route, based on a sequential word-by-word processing and the facilitated route, based on the prediction of complete structures, directly integrated to the situation model.

#### 6.4.1 Without facilitation: the classical compositional processing

This route corresponds to the classical architecture of language processing, relying on different levels of processing corresponding to the different domains: phonetics, phonology, morphology, syntax, and semantics. It occurs when no prediction/activation can be done from the situation model and corresponds to the more complex case, where no information can help in controlling or reducing the processing (for example, in the case of the interpretation of a complex text without context). In this case, the updating step consists in looking in the knowledge graph for slots where retrieved sign can be integrated. This mechanism is based on unification and resembles to those used during the lexical access. It consists in identifying all the slots in the graph with a free value (i.e., not already instantiated) and look for the best unification between the slot and the retrieved sign. By instantiating a slot, the information is propagated from the syntactic to the semantic structures due to structure sharing. This situation corresponds to the classical compositional view where a lexical processing is followed by a syntactic one and eventually a semantic interpretation.

#### 6.4.2 Facilitation: prediction and integration

Facilitation comes into play at two levels: prediction and integration (i.e., situation model updating). First, we have observed that when prediction reaches a certain probability level, the predicted sign can directly be used, without lexical access. Avoiding lexical access forms the first facilitation mechanism. The second facilitation level occurs when entire pieces of knowledge are predicted and integrated to the situation model. In this case, the predicted sign corresponds to sequences of words and complete knowledge subgraphs. In this case, updating integrates to the situation model a complete subgraph in a single step. This corresponds to the situation of a direct access to the meaning, without needing a compositional mechanism based on the syntactic structure. Typically, in the case of multi-word expressions (that can contain a large number of words), as soon as the expression is recognized, no syntactic processing is required anymore, the corresponding meaning being fully activated and integrated into the situation model. The third facilitation mechanism relies on activation. We have observed that the situation model graph contains activated signs, corresponding to information that has not been realized in the discourse (i.e., produced by the speaker) but with strong relationships with the context. Activated signs correspond to information that can be inferred and is then available for understanding. Moreover, the activation level plays a central role during updating: the most activated signs are used in priority when looking for a possible unification with the sign that has been produced by the speaker. This is a very efficient mechanism for reducing the search space and controlling unification.

To sum up, a complete facilitation situation occurs when a very predictable high-level sign, encoding a large set of information can be anchored with the speaker's production and integrated to the situation model with a highly activated sign. But note that this is not an all-or-nothing mechanism: it can be the case that the first facilitation (avoiding lexical access) is at work but not the last one (integration controlled by activation).

## 7 Discussion and perspectives

Most architectures and models of language processing have been built upon a restricted view of language limited to sentence processing. Studying language in its natural environment, typically conversation, needs to take into consideration the fact that the meaning is built by gathering in a very efficient manner different sources of partial information. Moreover, it is also necessary to explain the fact that in many cases, language processing remains very shallow and not sequential. These characteristics correspond to facilitation phenomena that need to be integrated into a unique architecture.

We have presented in this study such a model, taking first advantage of the fundamental role of **prediction** which becomes a core mechanism. The context under discussion during a conversation (or when reading a text) forms a complete knowledge base in which entities and concepts are inter-connected. This base offers the possibility to make predictions at any level, which is exactly what happens in the brain whatever its activity. These characteristics are implemented in our model by the fact that basic components are objects of any granularity: they can correspond to words, sequences of words, complete pieces of knowledge, or on the opposite underspecified structures. The main facilitation effect explaining the efficiency of language processing comes from this double characteristics: prediction is always at work and signs at any granularity can be predicted. Note that this last characteristic has deep consequences on the theory of language. All linguistic theories relies on a mechanism (derivation, constraint solving, etc.) aggregating linearly objects of categories at the same level. We propose instead to consider that the linguistic objects used as basic components can be of any granularity and do not correspond necessarily to a category of the same level. Unlike most linguistic theories, this means that the integration mechanism is no longer linear in the sense that structures at any level, not necessarily corresponding to a defined category, can be assembled.

The second important aspect of the model concerns the mechanism used at each step of the processing cycle: **unification** when comparing the predicted sign with the segment of the audio input, during lexical access, for updating the situation model. At each stage, unification plays a double role: verifying the compatibility of two structures and building a resulting structure merging both. The role played by unification can thus be very different. In the case of lexical access, unification is the controlling mechanism for identifying the matching entry. In the case of situation model updating, it implements a non-linear compositionality by integrating the current sign (of any granularity level) into a structure. Note that this operation is highly controlled by the fact that the activation mechanism reduces the number of possible sites where the current sign can be integrated.

In addition to these roles, unification offers the possibility to manipulate complex structures (the signs). These structures encode many different information, possibly coming from any domain (phonetics, syntax, semantics etc.), and structure sharing (which is a form of unification) ensures the relation between the features of these domains. In other words, the interaction between the different domains (or even the different modalities) only relies on sign unification and structure sharing. This represents a major theoretical shift: instead of considering domain interaction in terms of interaction rules between domains, it is directly implemented at the sign level. In this way, parallelism of language processing can be observed in a completely different way. In the original proposal, the parallel architecture is considered at the level of the domains and the interaction is specified by rules between them (Jackendoff, 2007; Huettig et al., 2022). On the opposite, the type approach we use in our model is based on complex signs integrating all sources of information. This approach can still be observed as parallel in the sense that no domain should pre-exist or have a more important role than another. The difference lies in the fact that the interaction is done at the sign level instead of the domain-specific level. It is interesting to note that this distinction is classical in multimodal computing, merging different sources of information (or the different modalities) can be done early or late. Our approach comes to an early fusion of the data where the classical domain-interaction approach comes to a late fusion. Note again that when the prediction of a high-level complex sign integrating all sources of information is not possible, a low-level mechanism is at work for accessing the meaning. In a parallel processing perspective, this comes to follow the domain-specific stream at the same time before integrating them to the meaning (see for example the M/G-stream model proposed in Baggio, 2021).

Unification of complex structures offers finally a very precise and efficient way to measure the distance between what is predicted and what is produced in the input (the *prediction error* in the predictive coding paradigm). In particular, due to the weights associated with the features and relations in a sign, it becomes possible to estimate precisely the level of the error and its source. When studying the brain correlates of language processing, it constitutes an efficient tool for estimating the type and the level of prediction error that can be correlated with the type and the amplitude of the response in the brain signal, for example, with event-related potentials. Moreover, in the case of mismatch, the importance of the failure can also be assessed due to the weights, leading to a possible constraint relaxation: such mechanism informs about the possibility of repairing the mismatch (possibly anticipating a late positivity in the brain signal).

This prediction-unification model provides in conclusion a framework bringing together into a unique architecture facilitation mechanisms besides a classical incremental processing. This is done due to a single processing cycle based on the integration of complex multi-level structures. In addition to explaining how to integrate facilitation mechanisms, this model also brings a new vision about the two different ways to build the meaning: compositionality or direct access. In our approach, these two mechanisms only differs from one point: the granularity of the signs to integrate into the situation model. Building the meaning is always done compositionally but can correspond to a word-by-word incremental mechanism (the classical view of compositional principle) or on the opposite in the integration of entire and large pieces of meaning.

## Data availability statement

The original contributions presented in the study are included in the article/supplementary material, further inquiries can be directed to the corresponding author.

## Author contributions

PB: Writing – review & editing, Writing – original draft, Visualization, Validation, Supervision, Software, Resources, Project administration, Methodology, Investigation, Funding acquisition, Formal analysis, Data curation, Conceptualization.

## Footnotes

^1^A common point of these theories is to get rid of production rules, replacing them by general principles and mechanisms due to this rich lexical encoding.

^2^Note that formally, predicates and signs (in other words attribute-value structures) both correspond to graphs.

^3^Note that even if we consider here the case of conversation, word segmentation also constitutes the preliminary stage in reading.
